# Efficacy and Safety of Shenqisuxin Granule for Non-ST-segment Elevation Acute Coronary Syndrome: Study Protocol for a Randomized, Double-Blinded, Placebo-Controlled Trial

**DOI:** 10.3389/fcvm.2022.888724

**Published:** 2022-06-09

**Authors:** Xiaoping Wu, Ming Guo, Shihua Shi, Shengnan Shi, Yanping Deng, Shenglan Wang, Yabing Wang, Peili Wang, Keji Chen

**Affiliations:** ^1^National Clinical Research Center for Chinese Medicine Cardiology, Xiyuan Hospital, China Academy of Chinese Medical Sciences, Beijing, China; ^2^Department of Geriatric, Hospital of Chengdu University of Traditional Chinese Medicine, Chengdu, China; ^3^Department of Epidemiology and Public Health, Swiss Tropical and Public Health Institute, Basel, Switzerland; ^4^Faculty of Science, University of Basel, Basel, Switzerland; ^5^School of Acupuncture-Moxibustion and Tuina, Beijing University of Chinese Medicine, Beijing, China; ^6^Department of Psychiatry and Medical Genetics, University of Alberta, Edmonton, AB, Canada

**Keywords:** Shenqisuxin granule, non-ST-segment elevation acute coronary syndrome, intestinal flora, randomized controlled trial, Chinese herbs

## Abstract

**Introduction:**

The Chinese herbal compound formula, Shenqisuxin granule (SQSX), promotes neovascularization and prevents in-stent restenosis in modern pharmaceutical studies and is expected to provide an effective strategy for non-ST-segment elevation acute coronary syndrome (NSTEACS). Thus, this study aims to examine the efficacy and safety of SQSX for NSTEACS and initially reveal its mechanism.

**Methods/Design:**

The study is a randomized, double-blinded and placebo-controlled trial. A total of 66 participants will be randomly allocated to one of the following two groups. Participants in the SQSX group will receive conventional treatment plus SQSX, while the placebo group will receive conventional treatment plus placebo, both for 14 days. The primary outcome, hs-CRP, and secondary outcome the Seattle Angina Questionnaire (SAQ) will be assessed at baseline, 7 ± 3 days and 14 ± 3 days. At all visit windows, other indicators including creatine kinase (CK), creatine kinase-myocardial band (CK-MB), cardiac troponins I (cTnI), 12-lead electrocardiograph and the syndrome scores of Qi deficiency and blood stasis will be tested and metagenomic sequencing for intestinal flora will be performed. Echocardiography and safety assessment will be performed at baseline and 14 ± 3 days. Adverse events will be monitored during the trial.

**Discussion:**

The purpose of the study is to examine the efficacy and safety of SQSX to improve NSTEACS and initially reveal its mechanism.

**Trial Registration:**

China Clinical Trial Registry, ChiCTR2000029226. Registered on January 19, 2020.

## Introduction

Non-ST-segment elevation acute coronary syndrome (NSTEACS) is the most common type of acute coronary syndrome (ACS) (1, 2). Despite proven benefits of leading-edge ACS salvage strategies, NSTEACS has a high 1-year main adverse cardiovascular and cerebrovascular events (MACCE) rate of 8.7%, and is associated with a higher risk of long-term mortality (3–5). Thus, it is vital to improve the secondary prevention of NSTEACS and reduce the incidence of MACCE.

High-sensitivity C-reactive protein (hs-CRP) is regarded as an important indicator of the incidence of MACCE. Systemic vascular inflammation plays multiple roles in the presentation of ACS, and hs-CRP is positively associated with the incidence of future MACCE (6–8). The Seattle Angina Questionnaire (SAQ) is widely utilized in the assessment of angina symptoms. The cTnI is an important biomarker to indicate the progression of acute myocardial ischemia. In addition, the disturbed intestinal flora is implicated in forming atherosclerotic plaques (9, 10). The alterations in the intestinal flora are correlated with coronary artery disease (CAD) severity via the mediation of intestinal flora serum metabolites (11). The level of trimethylamine-N-oxide (TMAO) in NSTEACS patients is significantly higher than that in healthy people and is positively correlated with the risk of MACCE (11, 12). Most studies focus on Chinese herbal compounds that act directly on target cells or organs (13). Emerging evidence indicates that Chinese herbal ingredients cannot be absorbed into the blood circulation directly through digestion (14–16). Therefore, the intestinal flora might be an important pathway for Chinese herbs to function.

The Chinese herbal compound formula, Shenqisuxin granule (SQSX) is a novel patented drug (Chinese patent number ZL202010122712.6) for CAD. It comprises six herbs: Huangqi (Astragali Radix), Danggui (Angelicae Sinensis Radix), Danshen (Salviae Miltiorrhizae Radix Et Rhizoma), Ezhu (Curcumae Rhizoma), Huanglian (Coptidis Rhizoma) and Baizhu (Atractylodis Macrocephalae Rhizoma). Based on our previous pharmaceutical studies, the major ingredients of SQSX can activate phosphatidylinositol 3-hydroxykinase-protein kinase B (PI3K-Akt) signaling pathyway and elevate the expression of VEGF mRNA in ischaemic myocardium of rats to promote neovascularization and angiogenesis (17). It can regulate the expression of Ang1/Tie2 and Ang2/Tie2 in hibernating myocardium and promote neovascularization and maturation to improve cardiac function (18). It's also proven that the ingredients of SQSX inhibit the proliferation of vascular smooth muscle cells in the in-stent restenosis minipig model induced by balloon injury, thereby preventing in-stent restenosis (19). At a time when the treatment of NSTEACS has run into a bottleneck, SQSX is expected to provide an effective strategy for further improvement of NSTEACS. Thus, this study aims to examine the efficacy and safety of SQSX for NSTEACS and initially explore its mechanism.

## Methods/Design

### Study Design

**Figure 1:**
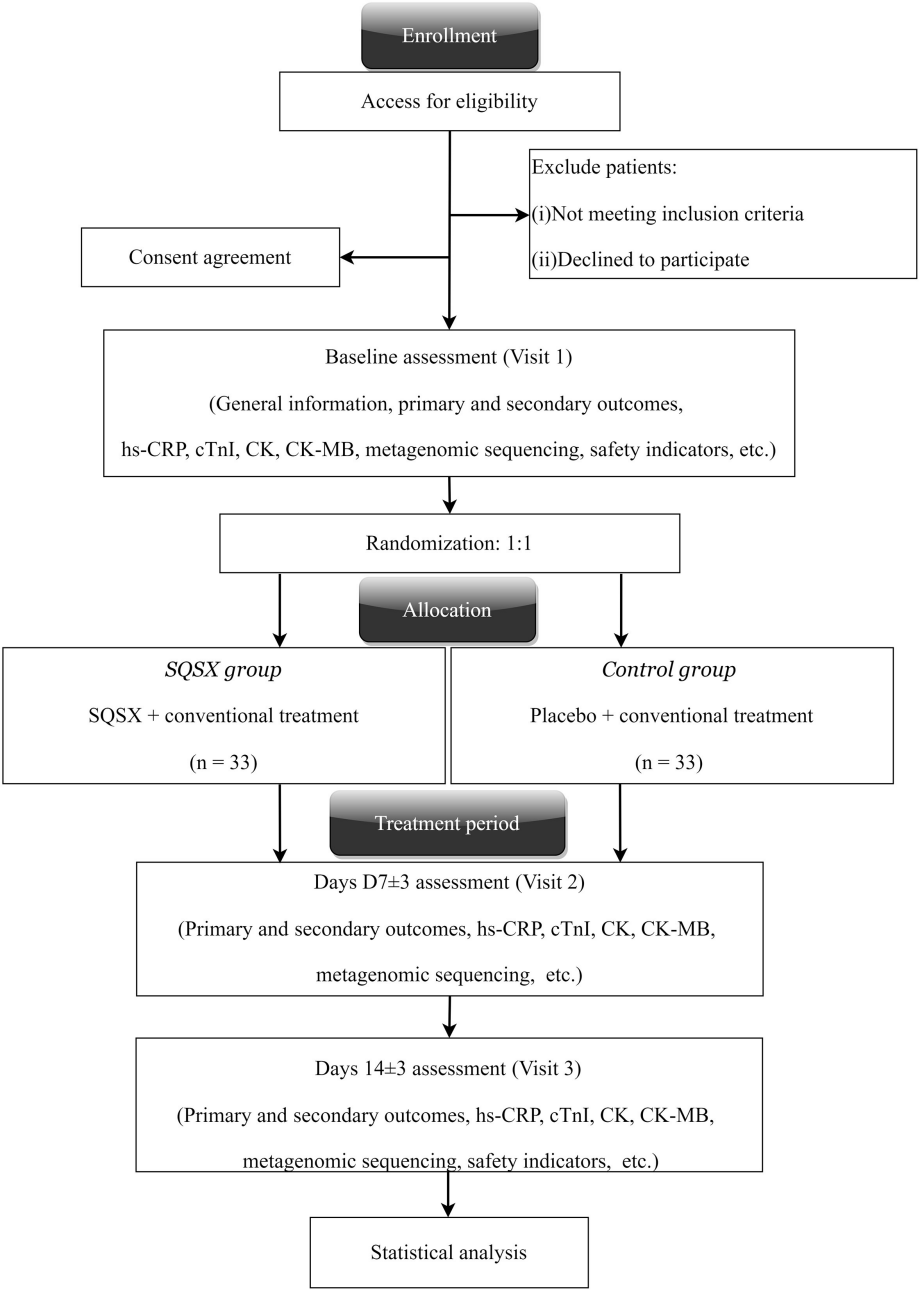
Study flowchart.

The randomized, double-blinded and placebo-controlled trial was endorsed by the Ethics Committee of Xiyuan Hospital, China Academy of Chinese Medical Sciences (Version No. XYYY-ASKY-1.1, January 1, 2021), and registered in the Chinese Clinical Trials Registry (ChiCTR2000029226). Our study conforms to the Helsinki Declaration and the Good Clinical Practice guidelines. It follows the Consolidated Standards of Reporting Trials Extension for Chinese Herbal Medicine Formulas 2017 (CONSORT-CHM Formulas 2017)recommendations and the Standard Protocol Items: Recommendations for Interventional Trials statements (SPIRIT) (20, 21). The study is currently underway at the Xiyuan Hospital, China Academy of Chinese Medical Sciences. Altogether 66 participants will be enrolled and randomly allocated to two groups. All participants will receive their respective interventions for 14 days. The clinical evaluations will be conducted at baseline (Visit 1), days 7 ± 3 (Visit 2) and 14 ± 3 (Visit 3), respectively. Figure 1 depicts a schematic of the study design.

### Participants

Specially trained research clinicians, the attending physician and general practitioners are responsible for the recruitment. Written informed consent is obtained for entry into the study.

### Inclusion Criteria

Subjects who meet all of the following criteria are eligible to participate in the study: (1) diagnosed as unstable angina (UA) or non-ST segment elevated myocardial infarction (NSTEMI) according to the 2015 ESC guidelines for the management of NSTEACS (22); (2) diagnosed as the TCM syndrome of Qi deficiency and blood stasis according to the Guiding Principles For Clinical Research Of New Chinese Medicines (23); (3) age 40–60 years; (4) heart function is Killip grade I-II for subjects diagnosed as NSTEMI; (5) in-hospital patients; (6) written informed consent is obtained.

### Exclusion Criteria

Any of the single conditions as follows would be excluded: (1) indication for renal insufficiency, blood serum creatinine (Scr) > 220μmol/l in males or Scr > 175 μmol/l in females; (2) significant abnormalities in liver function with alanine transaminase (ALT) and aspartate transaminase (AST) are 3 times above the upper bound of normal; (3) systolic pressure > 160 mmHg or diastolic pressure > 100 mmHg after controlled; (4) diabetic patients with random blood glucose ≥ 13.7 mmol/l or glycosylated hemoglobin ≥ 9.5%; (5) Women who are pregnant, breast-feeding or preparing to be pregnant; (6) combined with acute cerebrovascular disease,severe hematopoietic diseases, psychiatric disorder, or malignant tumor; (7) intestinal disease with inflammation or malabsorption; (8) participated in other clinical trials within 3 months; (9) GRACE score > 140 points (high risk group with 1~3% in-hospital death rate) (24); (10) had received antibiotics, steroids, laxatives, antidiarrheals or probiotics within the preceding 3 months.

### Withdrawal, Dropout, and Discontinuation

Patients have the right to exit the study at any time. Dropouts and reasons for leaving will be documented in the case report form (CRF). Data on these participants will also be incorporated into the statistical analysis. The reasons for discontinuing the trial by the principal investigator are: (1) participant experiences more clinical complications or a serious adverse event; (2) financial and management reasons; (3) administrative authorities terminate the trial.

### Intervention

All participants will be randomly allocated to one of the following two groups. Participants in the SQSX group will receive conventional treatment plus SQSX, while the placebo group will receive conventional treatment plus placebo, both for 14 days. The conventional treatment is based on the NSTEACS guideline issued by the European Heart Association in 2015 (22), including but not limited to the following: (1) anti-myocardial ischemic drugs: isosorbide mononitrate 20mg twice daily; (2) platelet inhibition: aspirin 100 mg once daily and ticagrelor 90 mg twice daily; (3) anticoagulation: enoxaparin 1 mg/kg s.c. twice daily; (4) invasive coronary angiography and revascularization according to the practical condition; (5) high-intensity statin therapy: atorvastatin 40 mg po qn. The composition and daily dosage of SQSX equal to Huangqi (Astragali Radix) 50 g, Danggui (Angelicae Sinensis Radix) 10 g, Danshen (Salviae Miltiorrhizae Radix Et Rhizoma) 30 g, Ezhu (Curcumae Rhizoma) 20 g, Huanglian (Coptidis Rhizoma) 10 g and Baizhu (Atractylodis Macrocephalae Rhizoma) 10 g. The placebo is made up of 5% granule and 95% dextrin with a look, smell and taste similar to active granule. Both SQSX and placebo (both 10 g per bag) are produced by Beijing Kangrentang Pharmaceutical Co., Ltd. (Beijing, China) in full accordance with the standards of Chinese Pharmacopeia and Good Manufacturing Practice. They use the same package with the study name and drug number printed on the surface. Subjects will be told to mix the granules with 100 mL of boiled water until dissolved and to drink it 30 min after breakfast and dinner. (10 g at a time, twice a day). To ensure adherence, study medicine will be distributed and/or retrieved at each visit.

During the study period, the use of any other Chinese medicine for the treatment of CAD is forbidden. Antibacterial drugs, probiotic drugs, proton pump inhibitors, immunosuppressants, hormones, drugs for gastrointestinal diseases, and drugs with a direct effect on intestinal flora are not permitted.

### Outcomes Measures

The primary outcome, hs-CRP, and secondary outcome the SAQ will be assessed at baseline, 7 ± 3 days and 14 ± 3 days. At all visit windows, other indicators including creatine kinase (CK), creatine kinase-myocardial band (CK-MB), cardiac troponins I (cTnI), 12-lead electrocardiograph and the syndrome scores of Qi deficiency and blood stasis will be tested and metagenomic sequencing for intestinal flora will be performed (25). Echocardiography and safety assessment will be performed at baseline and 14 ± 3 days. All adverse events will be tracked throughout the study. Table 1 lists the items measured as well as the data collection window.

**Table 1:** Data collection items and measurement points.

	**Screening**	**Treatment period**		
	**(Visit 0)**	**D1** **(Visit 1)**	**D7 ± 3** **(Visit 2)**	**D14 ±3** **(Visit 3)**
**Patients**				
Inclusion/exclusion criteria		×		
Informed consent	×			
General information ^a)^	×			
Medical and treatment history recording	×			
Randomization and allocation	×			
**Intervention**				
SQSX group		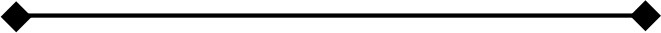		
Control group		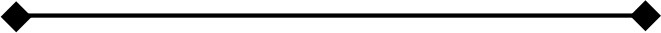		
**Outcomes**				
hs-CRP		×	×	×
SAQ		×	×	×
Syndrome scores of Qi deficiency and blood stasis (25)		×	×	×
cTnI	×	×	×	×
CK	×	×	×	×
CK-MB	×	×	×	×
Fecal collection and metagenomic sequencing		×	×	×
12-lead electrocardiograph	×	×	×	×
Echocardiography		×		×
Safety indicators ^b)^		×		×
**Others**				
Adverse events			×	×
Drug distribution		×	×	
Medicine recycling			×	×

a) *General information includes: name, birth, sex, nationality, marriage and work, etc*.

b) *Safety indicators include: complete blood count, liver and kidney function tests, routine urine and stool tests*.

### Adverse Events

Negative or unintended clinical medical effects that occur after treatment are known as AEs, which can be manifested as symptoms, signs, and abnormal clinical or laboratory findings. During the study, all related AEs details will be documented on the CRF. UA/NSTEMI is an urgent condition. Several complications including STEMI, heart failure, malignant arrhythmia and even sudden cardiac death may occur. Therefore, first aid measures will be available under guidelines and standards to ensure the safety of patients.

### Gut Microbial Profiling

Metagenomic sequencing will be used to analyze the spectrum of specific intestinal flora in NSTEACS patients after treatment with SQSX, revealing the mechanism of pharmacological effect from the intestinal flora perspective. The fecal collection kit and proper instruction for stool collection will be prepared for participants. Fecal samples will be stored in a laboratory freezer (−80°C) until metagenomic sequencing. The main steps of metagenomic sequencing are as follows: (1) genomic DNA is extracted and purified from feces using the QIAamp DNA kit; (2) genomic DNA is then fragmented to 350-bp by a Covaris crusher; (3) terminal repair, A-tailed, sequencing connector and other steps are performed to prepare a short cDNA library; (4) the library is then sequenced with Illumina Hiseq; (5) the sequence is assembled and finally analyzed for species and functional annotation.

### Randomization and Blinding

The randomization will be conducted by the Institute of Clinical Pharmacology of Xiyuan Hospital, China Academy of Chinese Medical Sciences. The SAS software-generated random sequencing in blocks of four was used for randomization. Eligible patients are randomized into the SQSX or control group on a 1:1 basis. Researchers and outcome evaluators involved in this study know nothing about the details of the randomization sequence. All patients and physicians will be unaware of treatment allocation until the end of the study. Urgent letters revealing group and treatment allocation have been prepared. If an emergency occurs, blind breaking and urgent measures may need.

### Data Management and Monitoring

All participant data on the CRF will be input into the clinical trial data management system. To ensure the authenticity, accuracy and completeness of data, several effective procedures are used. First, data cleaning will consider missing data, questionable values and inconsistent dates. After that, the source data will be verified by the supervisor to check for consistency. Third, manual checks. Clinical monitors will be appointed by the quality assurance department of Xiyuan Hospital, China Academy of Chinese Medical Sciences to undertake regular monitoring visits on the trial's progress and completion.

### Investigator Training and Quality Control

All investigators engaged in the study will be trained in standard operating procedure (SOP). The training includes screening of eligible subjects, standard collection and storage procedures for stool samples, and the use of evaluation scales, etc. All results will be reported by outcome evaluators trained in SOP standards to ensure their reliability and validity. In addition, to ensure relevant clinical data is collected as planned, researchers will establish close connection with patients through phone and WeChat groups.

### Sample Size Calculation

The sample size was estimated using hs-CRP. Currently, the hs-CRP of NSTEACS decreased from 5.30 mg/L to 3.74 mg/L after 14 days of conventional treatment, with a decline rate of 29.43% (26). The hypothesis is that the treatment group could reduce the hs-CRP of NSTEACS by 35%. With a type I error rate of α = 0.05 and a power of 80% (a type II error rate of β = 0.2), one arm requires a sample size of 27. With a 20% drop-out rate, we have to recruit a total of 66 patients. The following is the formula for calculating sample size (27):


$$\begin{array}{l} \text{n}=2{\left[\frac{\left({\text{u}}_{\alpha }+{\text{u}}_{\beta }\right)}{\delta /\sigma }\right]}^{2}+\frac{1}{4}u_{\alpha }^{2} \end{array}$$

n, is the sample size required in each group, δ = μ_1_-μ_2_, is the difference between means of two groups, σ, is the standard deviation, u_α_ and u_β_ are the values of u corresponding to the test level α and type II error probability β, respectively.

### Statistical Analyses

An independent biostatistician will perform statistical analysis. The result of recruitment, the specific reasons for data missing and the distribution of each statistical analysis data set will be described. The primary outcome, the change values of hs-CRP from recruitment to day 7 ± 3, day 14 ± 3 and the day of revascularization, and the secondary outcome, the change scores in five dimensions of SAQ at the same time, are both continuous variables. For comparison between groups, the continuous variables will be described using means ± SD, median, interquartile range, etc., and the Shapiro-Wilk normality test and Levene test for variance homogeneity will be performed. A Student *t* test will be used to compare two groups that have a normal distribution with homogeneous variance, otherwise the Wilcoxon rank-sum test will be used. When some continuous variables are converted into categorical variables, these categorical variables will be described using rates, and the chi-square test or Fisher's exact probability method will be used. For comparison within groups, the paired *t* test or Wilcoxon signed-rank test will be used for continuous variables and the McNemar test for categorical variables. If there are baseline imbalances, Cox regression analysis, logistic regression and multivariate analysis of covariance will be used to adjust confounding variables. The longitudinal data with repeated measures will be analyzed using the linear mixed-effects model to assess associations. The safety analysis will tabulate a detailed description of the adverse reactions, adverse events and serious adverse events and compare the difference in incidence between two groups using Fisher's exact probability method. A difference of *P* < 0.05 at two-tailed test will be considered statistically significant in all analyses. The statistical analyses will be performed using SAS software version 9.4 (SAS Institute, Cary, NC, USA).

After metagenomic sequencing is completed, microbiota community structures will be compared at different taxonomic levels. The abundance and diversity of intestinal flora will be analyzed. The relationship between microbiota composition and clinical outcomes will be investigated using univariate and multivariate linear and nonlinear mixed and regression models. Gene annotation will be conducted to study the function of the intestinal flora.

## Discussion

Based on our previous pharmaceutical studies, SQSX shows a good pharmacological effect of neovascularization and preventing in-stent restenosis. In clinical practice, it's also widely prescribed as a complementary medicine for CAD. Therefore, we would like to initiate a randomized, double-blind, placebo-controlled trial to examine the efficacy and safety of SQSX for NSTEACS. ACS is usually accompanied by gastrointestinal ulcers or bleeding (28, 29), and long-term use of antiplatelet drugs or combined use of multiple anticoagulant drugs causes gastrointestinal mucosal damage (30, 31). These pathological states of the intestinal mucosal barrier cause translocation and disturbance of intestinal flora (32). The disturbed intestinal flora and its metabolite TMAO are one of the reasons that promote the progression of CAD (12, 33, 34). As is well known, NSTEACS is at a critical stage from stable to unstable, which is likely to be accompanied by significant changes in the intestinal flora. We consider that SQSX could be an effective intestinal flora regulator, helping to ease the short-term symptoms of NSTEACS by improving intestinal flora disturbances and gastrointestinal tract injury. The study is possible to provide evidence for the use of intestinal flora modifiers in the treatment of ACS. The above hypotheses are still unclear, so we intend to verify in this trial.

There are three limitations to this work. First, the sample size of the study is small and might be biased. Therefore, two subsequent clinical studies with a larger sample size (*n* = 120) will be launched this year as a follow-up supplement. Second, the biomarker hs-CRP is observed as a surrogate indicator for the incidence of MACCE and does not yet fully explain the efficacy. We'll determine whether or not to investigate the incidence of MACCE further according to the result. Third, we only control the effects of combined medications on the intestinal flora, and could hardly standardize the patient's eating habits and other multivariate factors that affect intestinal flora. We will educate patients and document their dietary and lifestyle habits to ensure consistency of multiple factors as much as possible.

To conclude, the purpose of the trial is to examine the efficacy and safety of SQSX for NSTEACS and explore the possible pharmacological mechanisms.

### Trial Status

The trial was initiated in September 2020 and is presently recruiting participants. Currently, a total of 20 participants have completed the 14- days follow-up. However, no analysis has been conducted since the commencement of the trial.

## Data Availability Statement

The original contributions presented in the study are included in the article/supplementary material, further inquiries can be directed to the corresponding authors.

## Ethics Statement

The study conforms to the Helsinki Declaration. The study protocol was reviewed and approved by the Ethic Committee of Xiyuan Hospital, China Academy of Chinese Medical Sciences (2019XLA067-2). Written informed consent is obtained for entry into the study.

## Author Contributions

PW conceptualized and designed the study under the guidance of KC and XW drafted the manuscript, participated in the preliminary preparation of the study. MG reviewed the protocol for important intellectual content. XW and YD are responsible for the execution of the clinical trials. ShiS, SheS, SW, and YW helped to polish the manuscript. All authors read and approved the final manuscript.

## Funding

This trial was supported financially by the National Natural Science Foundation of China (No. 81973681).

## Conflict of Interest

The authors declare that the research was conducted in the absence of any commercial or financial relationships that could be construed as a potential conflict of interest.

## Publisher's Note

All claims expressed in this article are solely those of the authors and do not necessarily represent those of their affiliated organizations, or those of the publisher, the editors and the reviewers. Any product that may be evaluated in this article, or claim that may be made by its manufacturer, is not guaranteed or endorsed by the publisher.
